# CHANGES IN PERSONAL RESOURCES AND SUBJECTIVE AGE ACROSS 8 YEARS

**DOI:** 10.1093/geroni/igz038.1701

**Published:** 2019-11-08

**Authors:** Allura F Lothary, Thomas M Hess

**Affiliations:** North Carolina State University, Raleigh, North Carolina, United States

## Abstract

Subjective age has been extensively researched as a predictor of physical and mental health outcomes in older adulthood. However, the mechanisms behind subjective age have not yet been established and reasons for longitudinal changes are unclear. Selective engagement theory (SET; Hess, 2014) proposes a connection between increases in objective and subjective cognitive costs and motivation to engage future resources. Costs are influenced by personal resources, mainly physical and mental health. These changes in costs may also influence subjective experiences of the aging process. For example, aging attitudes have been shown to partially mediate the associations between resources and both motivation and activity engagement (Hess et al., 2018). Using the Health and Retirement Study (HRS) data, changes in resources (physical and mental health) were hypothesized to predict changes in subjective age across 8 years. More specifically, reductions in resources were predicted to be associated with older subjective ages over time. This relationship was expected to be mediated through aging attitudes. Results indicate an association between changes in resources and changes in subjective age over time in the expected direction. Changes in resources were partially mediated by aging attitudes. However, resources function differently in predicting changes in subjective age based on the resource (physical and mental health) and chronological age of the participant. These findings provide more guidance as to the mechanisms behind subjective age and may offer theoretical guidance for future work looking at understanding changes in subjective age.

